# Blue Exciplexes in Organic Light-Emitting Diodes: Opportunities and Challenges

**DOI:** 10.3390/molecules30071556

**Published:** 2025-03-31

**Authors:** Duxu Yan, Mengmeng Zhang, Jintao Wang, Xiaoqing Jing, Jun Sun, Yongan Zhang, Liping Yang, Ren Sheng, Ping Chen

**Affiliations:** 1 Institute of Physics and Electronic Information, Yantai University, Yantai 264005, China; yandx316@s.ytu.edu.cn (D.Y.); zmm20011011@163.com (M.Z.); 17616180805@s.ytu.edu.cn (X.J.); ytdxjunsun@s.ytu.edu.cn (J.S.); yonganzhang@s.ytu.edu.cn (Y.Z.); 2 School of Information Engineering, Yantai Institute of Technology, Yantai 264005, China; yanglp202410@163.com

**Keywords:** blue exciplex, OLED, TADF, efficiency, blue emission, host materials

## Abstract

Blue exciplexes, a critical innovative component in organic light-emitting diodes (OLEDs) technology, exhibit substantial potential for enhancing device efficiency, reducing driving voltage, and simplifying structural designs. This article reviews the pivotal role of blue exciplexes in OLEDs, analyzing their unique advantages and challenges as emitters and host materials. Through optimized molecular design, blue exciplexes achieve high color purity and emission efficiency, surpassing conventional fluorescent materials. Additionally, their wide energy bands and high triplet energy provide opportunities to improve the performance of sky-blue, deep-blue, and white OLEDs. However, limitations in deep-blue efficiency, material degradation due to high-energy excitons, and spectral red-shift pose significant challenges to their development. This review offers a comprehensive perspective and research reference on the photophysical mechanisms of blue exciplexes and their applications in display and lighting fields.

## 1. Introduction

Over the past 30 years, significant progress has been made in the research of organic light-emitting diodes (OLEDs) [1,2,3,4,5,6,7]. Since 1987, after Eastman Kodak and Tang’s team fabricated the first highly efficient, low-voltage OLED device, the research and development of OLED technology have advanced rapidly [3,4]. To overcome the limitation of traditional fluorescent excitons, which exhibit a utilization efficiency of merely 25%, S. R. Forrest and coworkers introduced phosphorescent emitting materials, achieving an internal quantum efficiency (IQE) of up to 100%. However, the frequent use of precious metals has constrained their further development [8,9]. In 2012, Adachi and colleagues first reported thermally activated delayed fluorescence (TADF) materials, which have since been recognized by both academia and industry as next-generation emitters. These materials feature a small singlet-triplet energy gap (ΔE_ST_), enabling triplet excitons to undergo reverse intersystem crossing (RISC) to singlet excitons at room temperature, thereby generating delayed fluorescence [10,11]. In the same year, they reported an exciplex system and, by introducing the concept of TADF, elucidated the formation mechanism and energy transfer processes of the exciplex system, laying the foundation for subsequent extensive research [12]. This finding highlights the substantial potential of exciplex-based systems in high-performance optoelectronic devices.

Among them, blue exciplexes have garnered significant attention due to their potential applications in high-color-purity displays and energy-efficient lighting technologies. However, their development and practical implementation face exceptional complexities compared to exciplexes emitting at longer wavelengths, primarily due to the demanding requirement of high excited-state energy (typically ≥2.8 eV) for blue emission [13,14,15,16]. The core challenges and unique characteristics of blue exciplex systems are outlined as follows: (i) exciplex emission tends to red-shift and broaden, making it challenging to sustain blue emission (420–490 nm) or achieve ideal Commission Internationale de l’Éclairage (CIE) coordinates (The National Television System Committee (NTSC) standard specifies that the CIE y-coordinate of deep-blue light should be ≤0.08) [17]; (ii) High triplet energy risks back-energy transfer or poor exciton confinement, reducing efficiency and stability [18,19]; and (iii) high-energy excitons degrade materials, shortening lifetimes [20].

Notwithstanding these challenges, blue exciplexes hold immense promise for realizing OLED devices with superior color purity and low driving voltage [13,21,22,23,24]. This review aims to systematically summarize the research progress and challenges in blue exciplex-based OLEDs, explore their future development pathways, and provide references and inspiration for researchers in this field. To enhance readability, the article is structured into three key sections: (1) fundamental theories of blue exciplex formation and emission mechanisms, (2) advances in blue exciplex systems as emitters and their applications in white organic light-emitting diodes (WOLEDs); and (3) advances in blue exciplexes as host materials and their applications in WOLEDs. Finally, the future prospects for the development of blue exciplex systems in OLEDs are discussed.

## 2. Photophysical Mechanisms of Blue Exciplexes

Similar to the formation mechanisms of exciplexes emitting other colors, the generation of blue exciplexes also involves an intermolecular charge transfer (CT) process, which occurs exclusively under excited-state conditions [25,26,27,28]. When one molecule (e.g., acceptor A or donor D) absorbs energy and transitions to an excited state, exciplexes (denoted as (A^δ−^D^δ^⁺)^*^) emerge through intermolecular collisions and orbital interactions. Subsequently, these exciplexes undergo radiative decay to return to the ground state, accompanied by photon emission.

Similarly, blue exciplexes are also accompanied by typical exciplex spectral characteristics such as red-shift and broadening [29,30,31,32,33]. This presents the initial challenge for blue exciplexes: selecting shorter-wavelength donors and acceptors to ensure pure or deep-blue emission despite spectral red-shift and broadening. The formation of exciplexes requires a thermodynamic driving force (−ΔGcs), making the selection of appropriate donor and acceptor materials critical. This driving force is typically quantified using a modified Rehm-Weller equation:

(1) $$-\Delta G_{cs}=E_{exciton}(E_{D}^{*}\;or\;E_{A}^{*})-E_{exciplex}$$

Here, E_exciton_ represents the exciton energy of constituent molecules (i.e., the excited-state energy of either the donor or acceptor), while E_exciplex_ denotes the exciplex energy. The exciton energy is assumed to be the energy difference between the HOMO of the donor and the LUMO of the acceptor, which can be estimated via electrochemical methods. Generally, the ideal condition for exciplex formation requires a driving force greater than 0.57 eV [34].

Furthermore, the triplet energy of an exciplex generally needs to be lower than that of its constituent molecules; otherwise, it may transfer to the molecular triplets via triplet-triplet energy transfer (TTET), thereby disrupting the reverse intersystem crossing (RISC) process [35,36]. The reverse intersystem crossing (RISC) process is fundamentally governed by its rate K_RISC_, which is critically dependent on the ΔE_ST_. The temperature-dependent RISC rate can be mathematically expressed as:

(2) $$K_{RISC}=Ae^{\left(-\frac{\Delta E_{ST}}{K_{B}T}\right)}$$

where A represents the pre-exponential factor, K_B_ is the Boltzmann constant, and T denotes the absolute temperature. This formulation reveals that K_RISC_ exhibits an exponential relationship with both ΔE_ST_ and T. In practice, with ambient temperature (T) often limited, minimizing ΔE_ST_ through molecular design is crucial for boosting RISC efficiency. A smaller ΔE_ST_ offers two key benefits: (i) faster RISC dynamics reduces the thermal activation barrier, enhancing the up-conversion of triplet excitons to singlets; (ii) higher RISC efficiency amplifies TADF, increasing the photoluminescence quantum yield (PLQY) [10,11,20,37,38].

**Figure 1:**
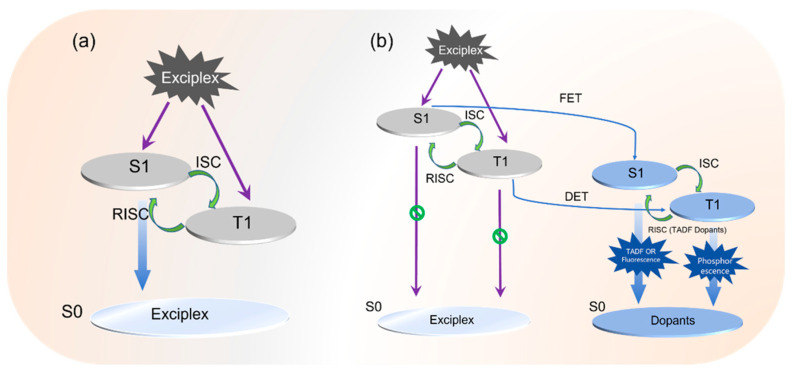
Mechanism diagrams of (**a**) the exciplex composite as an emitter and (**b**) as a host.

Moreover, in wide color gamut standards (e.g., DCI-P3 or Rec. 2020), the purity and intensity of blue light directly influence the vividness and authenticity of the displayed image. As an emitter, the blue exciplex composite, with its inherently high exciton utilization efficiency, holds the potential to replace traditional fluorescent materials. The emission mechanism is illustrated in Figure 1a. Furthermore, the shorter wavelength of blue exciplex composites corresponds to significantly higher photon energy compared to green (~2.3 eV) and red (~2.0 eV). Consequently, when used as a host, the blue exciplex composite is well-suited to accommodate dopants of nearly all colors. Moreover, the presence of RISC enables more efficient exciton utilization and energy transfer, with the underlying mechanism depicted in Figure 1b.

**Figure 2:**
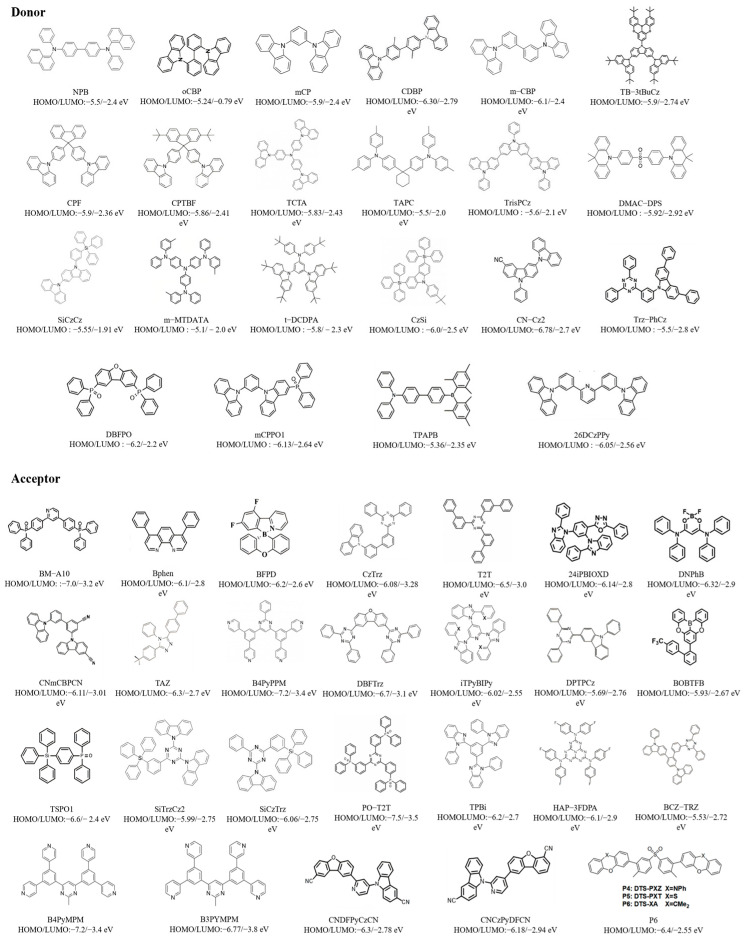
The chemical structures of all donors and acceptors of the exciplexes mentioned in this review and their HOMO/LUMO levels.

Simultaneously, the advancement of blue exciplex systems is inseparable from the development of donor and acceptor materials. Figure 2 presents the chemical structures of all donors and acceptors of the exciplexes mentioned in this review, along with their corresponding HOMO and LUMO energy levels.

## 3. Blue Exciplex as the Emitter in OLEDs

Blue exciplexes, when used as emitters, enable the fabrication of highly efficient blue OLED devices. Moreover, utilizing their blue emission properties, these exciplexes can be combined with yellow, green, or red phosphorescent or fluorescent dyes, facilitating the development of high-performance white OLED devices. This dual application demonstrates the versatility of blue exciplexes, offering the possibility for creating efficient blue emitting devices while also opening new pathways for the advancement of next-generation white OLEDs.

### 3.1. Exciplex as an Emitter in Blue OLEDs

**Figure 3:**
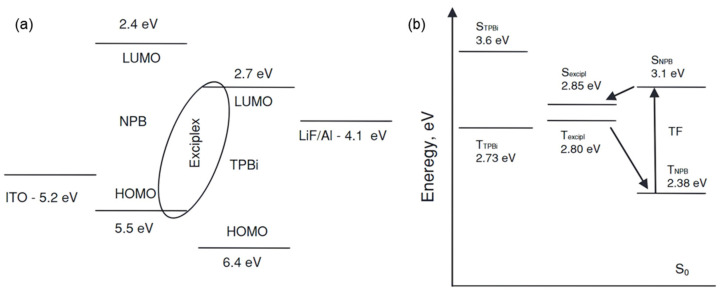
(**a**) Energy-level diagram of the NPB: TPBi exciplex; (**b**) schematic diagram of the TTF mechanism proposed by Jankus et al. [35].

Using exciplexes as emitters and efficiently harvesting triplet excitons significantly enhance the external quantum efficiency (EQE) of OLEDs. In 2013, Jankus et al. proposed a deep-blue exciplex with NPB (N,N’-Bis(naphthalen-1-yl)-N,N’-bis(phenyl)-benzidine) as the donor and TPBi (1,3,5-Tris(1-phenyl-1Hbenzimidazol-2-yl)benzene) as the acceptor. As an emitter, the exciplex achieved an external quantum efficiency of 3.7%, and electroluminescence at 450 nm. It is noteworthy that with the triplet energy level of NPB being lower than that of the exciplex, no TADF phenomenon existed in this exciplex. Jankus et al. explained the delayed fluorescence observed in Time-Resolved Photoluminescence (TRPL) through triplet-triplet fusion (TTF). As shown in Figure 3, they pointed out that it is precisely due to TTF that the exciplex exhibited additional EQE, demonstrating the potential of TTF in fabricating high-performance deep-blue OLEDs [35]. In the same year, Kim et al. reported a blue exciplex formed by TCTA (4,4’,4-Tris(carbazol-9-yl)triphenylamine) and B3PYMPM (4,6-Bis(3,5-di(pyridin-3-yl)phenyl)-2-methylpyrimidine). They observed that this exciplex exhibited pronounced delayed fluorescence, with the delayed emission increasing as the temperature decreased. At an extremely low temperature of 35 K, the exciplex achieved a photoluminescence (PL) efficiency of up to 100% and demonstrated efficient RISC rates ranging from 3 × 10^−6^ to 3 × 10^−4^ s^−1^. These findings suggest that the material possesses a minimal energy gap, enabling the RISC process from the triplet state to the singlet state without requiring thermal activation [39].

**Figure 4:**
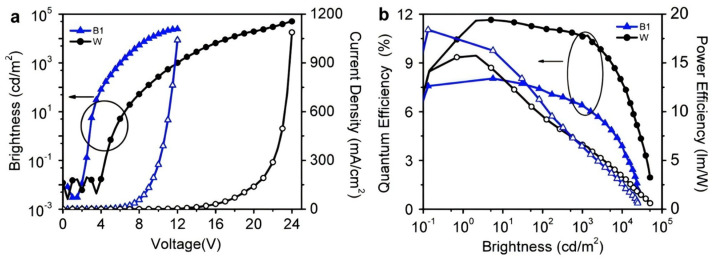
(**a**) Current density−voltage−luminance (J-V-L) characteristics and (**b**) external quantum efficiency−luminance−power efficiency (EQE-L-PE) characteristics of the mCP:PO-T2T exciplex (blue curve) reported by Hung et al. [40].

In 2014, Hung et al. reported a highly efficient blue exciplex emission phenomenon. The authors designed and synthesized a novel electron transport material called 2,4,6-Tris [3-(diphenylphosphinyl)phenyl]-1,3,5-triazine (PO-T2T), with HOMO and LUMO energy levels of −2.83 eV and −6.83 eV, respectively. The authors created a blue light-emitting device using an exciplex formed by (1,3-Bis(carbazol-9-yl)benzene) mCP: PO-T2T as the emitting layer. The device achieved an impressively low turn-on voltage of only 2 V and an EQE_max_ of 8% (Figure 4). The authors studied the emission mechanism and concluded that balancing electrons and holes in the device was crucial. They also highlighted the importance of ensuring sufficient contact between donor and acceptor molecules in the exciplex-doped layer to improve device efficiency [40]. In 2015, Liu et al. developed the NPB:DPTPCz exciplex emitter by blending the hole-transporting material N,N′-bis(1-naphthalenyl)-N,N′-bisphenyl-(1,1′-biphenyl)-4,4′-diamine (NPB) with the high-triplet-energy (T1) bipolar molecule 3-(4,6-diphenyl-1,3,5-triazin-2-yl)-9-phenyl-9H-carbazole (DPTPCz) in a 1:1 weight ratio. The normalized EL spectrum of the exciplex revealed an emission peak at 491 nm, and the resulting device achieved an EQE_max_ of 0.6%. This finding indicates that the NPB:DPTPCz exciplex exhibited some emissive capability in the blue spectral region. However, its low EQE suggests that both exciton utilization efficiency and device performance require further optimization [28].

In 2015, T. Zhang et al. reported the development of a highly efficient blue exciplex system exhibiting TADF characteristics, employing mCBP (3,3-di(carbazolyl)biphenyl) as the donor material and PO-T2T as the acceptor material. The exciplex emission peaked at 473 nm, and a 50 mol% mCBP:PO-T2T doped film demonstrated a photoluminescence quantum yield (PLQY) of 34 ± 4%. The optimized blue OLED achieved CIE coordinates of (0.17, 0.23), with a maximum EQE of 7.66%, a maximum CE of 15.08 cd/A, and a maximum power efficiency (PE) of 17.78 lm/W [41]. In the same year, Lee et al. developed a thermally activated delayed fluorescence (TADF) blue exciplex emitter by blending the hole-transporting molecule 4,4’-bis(9-carbazolyl)-2,2’-dimethylbiphenyl (CDBP) with the electron-transporting molecule ((1,3,5-triazine-2,4,6-triyl)tris(benzene-3,1-diyl))tris(diphenylphosphine oxide) (PO-T2T) in a 1:1 weight ratio. The resulting CDBP:PO-T2T blend exhibited a photoluminescence (PL) peak at 476 nm. Devices fabricated with this blue exciplex emitter achieved an impressive EQE_max_ of 13% and a PE_max_ of 27.8 lm/W, highlighting its potential for efficient blue OLED applications [34]. In 2019, Marian Chapran et al. explored non-doped exciplex blends utilizing PO-T2T as the electron acceptor, paired with CzSi, mCP, and mCPPO1, to investigate their emission properties and potential in OLED applications [42]. In device applications, the blue-emitting OLEDs based on CzSi:PO-T2T, mCP:PO-T2T, and mCPPO1:PO-T2T achieved maximum EQEs of 6.1%, 16%, and 6.5%, respectively, with mCP:PO-T2T exhibiting superior performance due to its minimal ΔE_ST_ and high TADF efficiency. The study suggests these exciplexes hold potential for multicolor OLEDs, achieving efficient emission without additional host materials, though color purity requires further optimization for display applications.

**Figure 5:**
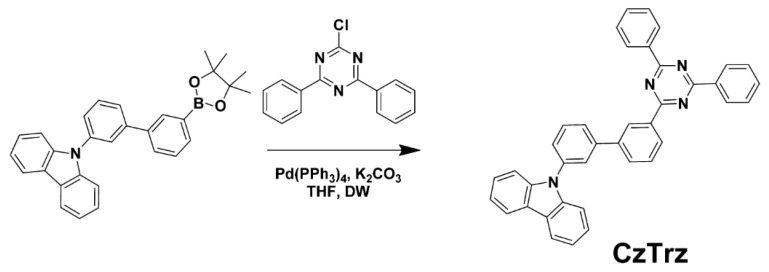
Synthetic scheme of CzTrz [43].

Although exciplex-based devices formed by mixing suitable donor and acceptor materials can achieve high EQE, further discussion is needed on strategies to enhance their performance. Exploring the molecular design of exciplex donor and acceptor materials is currently the key to improving the EQE of these devices. In 2015, Lee et al. developed a high-efficiency blue exciplex device by designing the acceptor material of 3′-(4,6-diphenyl-1,3,5-triazin-2-yl)-(1,1′-biphenyl)3-yl)-9-carbazole (CzTrz) with a suitable donor segment (Figure 5). The blue exciplex device, with TCTA as the donor and CzTrz as the acceptor, achieved a record-breaking EQE of 12.6%, which was the highest EQE reported for blue exciplex emitters at that time [43].

**Figure 6:**
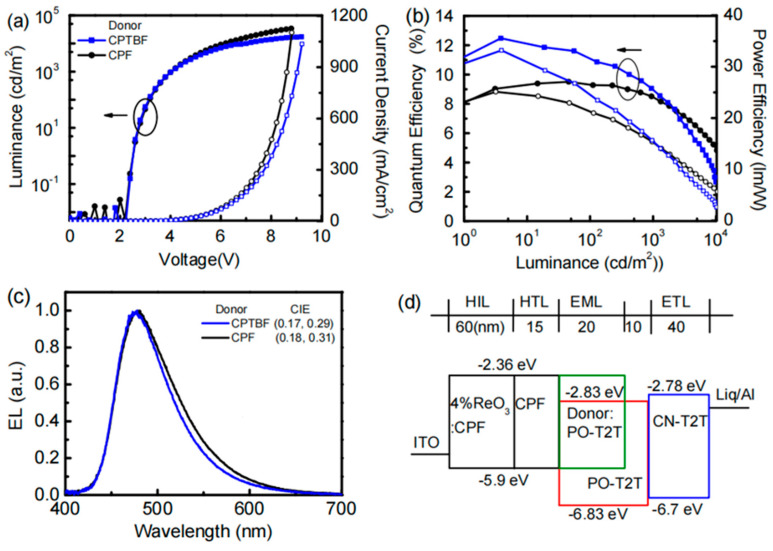
(**a**) Current density−voltage−luminance (J-V-L) characteristics, (**b**) EQE and PE as a function of luminance, (**c**) EL spectra, (**d**) device structure of blue exciplex devices using CPTBF and CPF as donor [44].

In 2017, Hung et al. first designed a carbazole-based electron donor molecule, 9,9-bis[4-(9-carbazolyl)phenyl]fluorene (CPF), which was combined with PO-T2T as the acceptor to form an exciplex. In Figure 6, the device achieved an EQE of 9.5%, a CE of 35.3 cd/A, and a PE of 41.3 lm/W. To further enhance device performance, the authors designed a tert-butyl group-substituted donor molecule, 9,9-bis[4-(carbazol-9-yl)phenyl]-2,7-di-tert-butylfluorene (CPTBF), to replace CPF as the exciplex donor. By introducing the remote steric effect, the effective contact area between donor and acceptor molecules was increased. As a result, the EQE_max_ of the blue exciplex device was improved to 12.5%. This proposed concept of the remote steric effect provides a novel and effective strategy for designing high performance exciplex-based OLED devices [44].

**Figure 7:**
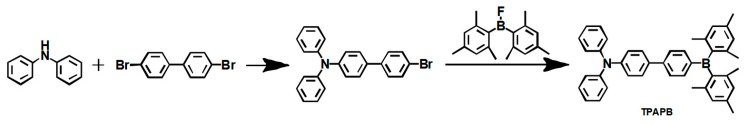
Synthetic route and chemical structure of TPAPB [45].

In 2015, Z. Chen et al. reported an OLED based on a highly efficient blue exciplex exhibiting TADF phenomenon. The research team designed and synthesized a novel donor material, TPAPB ((4-dimethylboryl)phenyltriphenylamine), with a LUMO level of 2.35 eV and a HOMO level of 5.36 eV (Figure 7). Using TPAPB as the donor and TPBi as the acceptor, the device achieved a maximum PE_max_ of 7.2 ± 0.5 lm W^−1^ and an EQE_max_ of 7.0 ± 0.4%. The authors attributed this excellent EQE to the high PLQY of the TPAPB:TPBi doped film and the enhanced efficiency resulting from the exciplex formation, which broadened the exciton recombination zone and improved the balance of hole and electron transport [45].

**Figure 8:**
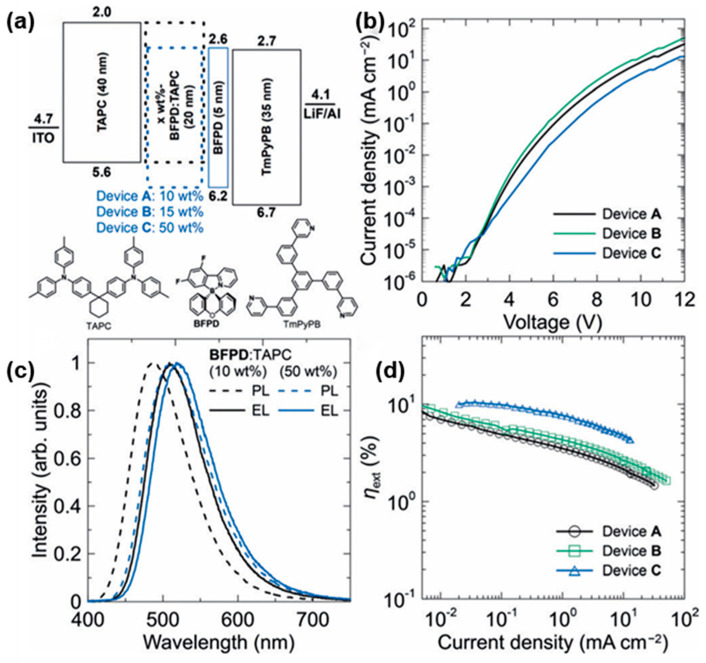
(**a**) Energy-level diagram of OLEDs using BFPD: TAPC as the emitting layer. (**b**) Current density−voltage (J-V) characteristics of the OLED devices. (**c**) PL spectra for 10 wt% and 50 wt% BFPD: TAPC films and EL spectra for devices A and C. (**d**) External electroluminescence quantum efficiency of the OLEDs as a function of current density [46].

In 2018, Adachi et al. proposed enhancing exciplex PLQY by studying excited-state dynamics. They designed and synthesized a spiro-type four-coordinate boron derivative, BFPD, as the exciplex acceptor and paired it with Di-[4-(N,N-di-p-tolyl-amino)-pheny l]cyc lohexane (TAPC) as the donor. As can be seen in Figure 8, when the donor-to-acceptor ratio of the exciplex was 1:1, the device exhibited optimal efficiency, achieving an EQE of 10.5%. This enhancement was attributed to the excellent energy level alignment of the BFPD: TAPC exciplex, which led to a substantial increase in delayed fluorescence under electrical excitation, indicating efficient triplet harvesting [46]. In the same year, Lin et al. synthesized a bipolar transport material with excellent thermal and morphological stability called CN-Cz2, which facilitated efficient energy transfer and improves OLED performance. The authors used PO-T2T as the exciplex acceptor, and the resulting CN-Cz2: PO-T2T thin film exhibited a PLQY of 55% in air. The resulting blue exciplex OLED achieved an excellent CE of 37.8 cd/A and PE of 47.5 lm/W [47]. In 2019, Tao et al. designed and synthesized two N-linked benzoimidazole/oxadiazole hybrid electron acceptors, 2-(2,4-bis(2-phenyl-1H-benzo[d]imidazol1-yl)phenyl)-5-phenyl-1,3,4-oxadiazole (24iPBIOXD) and 2-phenyl-5-(2,4,6-tris(2-phenyl-1H-benzo[d]imidazol-1yl)phenyl)-1,3,4-oxadiazole (iTPBIOXD), via a simple catalyst-free C-N coupling reaction. When paired with the electron donor mCP, the resulting deep-blue exciplex achieved EQEs of only 0.65% and 0.63%. The study found that the electroluminescent (EL) performance of blue exciplexes was closely tied to the HOMO-LUMO energy offset, with the poor emission performance of this deep-blue exciplex attributed to an insufficient energy offset [48].

**Figure 9:**
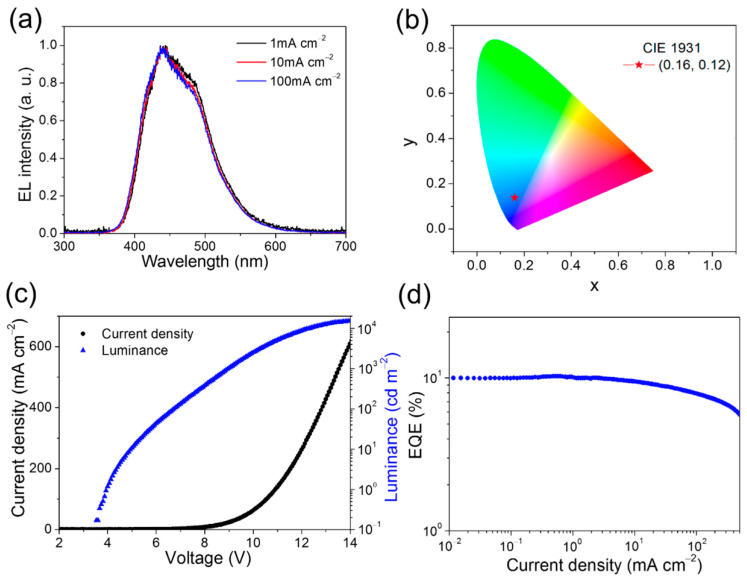
(**a**) The EL spectra of the 8 wt% HAP-3FDPA: mCP exciplex−based OLED at various current densities. (**b**) The deep-blue CIE coordinates of (0.16, 0.12) for EL spectra. (**c**) The current efficiency−voltage−luminance (J-V-L) characteristics. (**d**) EQE as a function of current density [24].

With the advancement of OLED technology, the design and synthesis of deep-blue emitting materials face numerous challenges, particularly due to the significant red-shifted and spectral broadening that often occur in exciplex emission compared to single compounds. In 2021, Li et al. proposed a novel exciplex system to achieve more desirable deep-blue emission characteristics. In this work, the authors designed a new material, 2,5,8-tris(di(4-fluorophenyl)amine)-1,3,4,6,7,9,9b-heptaazaphenalene (HAP-3FDPA) as an electron donor by incorporating three bis(4-fluorophenyl)amine moieties onto a heptazine backbone, and using mCP as the electron donor. The 8 wt% HAP-3FDPA: mCP exciplex system displayed a deep-blue emission with a peak wavelength of 433 nm and a high PLQY of 53.2%. As shown in Figure 9, an OLED containing this exciplex system as an emitting layer showed deep-blue emission with the peak EQE of 10.2%, and showed deep-blue CIE coordinates of (0.16, 0.12). The article highlighted that the small ∆E_ST_ facilitated efficient exciton up-conversion and minimized overlap between the HOMO and LUMO [49]. In 2023, Xie et al. developed an efficient deep-blue emitter TB-3tBuCz, and significant TADF behavior emerged when it was doped with the host material 2,6-Bis(3-(9H-carbazol-9-yl)phenyl)pyridine (26DCzPPy), leading to the formation of exciplex species. This process facilitated efficient spin-flip of triplet excitons, resulting in an EQE_max_ of 14.6% and deep-blue emission with CIE (0.158, 0.052) [50]. In the same year, Fu et al. developed the (3,30-di(9H-carbazol-9-yl) biphenyl) mCBP: DNPhB (difluoroboron(Z)-3-(diphenylamino)-3-hydroxyN,N-diphenylacrylamide) exciplex, which exhibited deep-blue emission at 444 nm. A deep-blue OLED fabricated with this exciplex emitter achieves an EQE_max_ of 4.83%, CIE coordinates of (0.152, 0.075). This high-performance wide-bandgap exciplex highlights its potential in deep-blue OLEDs, offering new opportunities for enhancing color purity and efficiency [22]. Furthermore, the NPB:TAZ exciplex proposed by K. N. Narayanan Unni et al. achieved deep-blue emission at 440 nm with a turn-on voltage of 3.6 V [51]. In 2025, Liu et al. designed a deep-blue fluorescent emitter, BOBTFB, which, when used as an acceptor paired with TCTA, achieved blue emission at 469 nm with CIE coordinates of (0.15, 0.18) [52].

**Figure 10:**
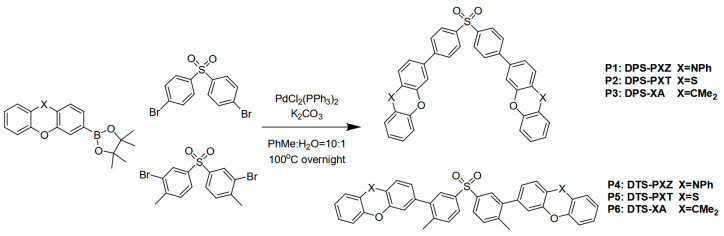
Synthetic routes for compounds P1–P6 [53].

Compared to bulk exciplexes, interface exciplexes can utilize doping-free technology, significantly simplifying the device structure. Due to the large energy level offset between the HOMO of the donor and the LUMO of the acceptor, charge carriers predominantly recombine at the heterojunction interface to form excitons for light emission. To ensure efficient emission from the interface exciplex, the triplet energy level (T1) of the exciplex should be lower than those of the donor or acceptor, preventing reverse energy transfer from the exciplex to the constituent molecules. To develop high-T1-energy-level materials for forming interface exciplexes, researchers have extensively explored molecular structures in recent years. In 2020, Tan et al. developed a material named P6, which had a T1 energy of 3.0 eV. P6 was shown to form an efficient interface exciplex with TCTA, enabling high-performance blue emission. In the TCTA: P6-based OLED, TCTA acted as the donor for exciplex formation, while P6 served as the acceptor (Figure 10). The exciplex-based OLED achieved an EQE_max_ of 9.1% and an impressive deep-blue emission at 433 nm, highlighting the potential of tuning interface exciplexes to enhance device performance. Furthermore, the authors predicted that similar device architectures could lead to further advancements in both blue OLEDs and WOLEDs, offering significant improvements in efficiency and emission characteristics [53].

In addition to developing suitable acceptor and donor materials to form exciplexes, researchers have proposed a strategy of employing materials with TADF characteristics as either the donor or acceptor in exciplex systems. The advantage lies in the fact that TADF-type donor or acceptor materials possess an intramolecular RISC process. Upon forming an exciplex, an additional intermolecular RISC process emerges. This dual RISC mechanism facilitates the up-conversion of triplet excitons into singlet excitons, thereby significantly enhancing the utilization efficiency of singlet excitons. In 2016, Li et al. investigated exciplex emitters formed by the bipolar material DMAC-DPS, a D-A-D-type molecule comprising a diphenylsulfone (DPS) acceptor and 9,9-dimethyl-9,10-dihydroacridine (DMAC) donor, paired with the acceptors T2T and B4PyMPm. Devices utilizing DMAC-DPS:T2T and DMAC-DPS:B4PyMPm blends as the emitting layer exhibited exciplex emission peaks at 480 nm and 493 nm, respectively. The blue and sky-blue devices achieved external quantum efficiencies (EQE) of 4.44% and 4.40%, respectively, demonstrating that the combination of DMAC-DPS as a donor with different acceptors could effectively tune the emission wavelength of exciplex systems, offering a reference for the development of blue OLEDs, though their performance still has room for improvement [54]. In 2020, T. B. Nguyen et al. explored the luminescent properties of a blue exciplex system comprising TrisPCz as the donor and BCz-TRZ as the acceptor [55]. Notably, BCz-TRZ is an acceptor material endowed with TADF properties. Their findings revealed that the TrisPCz:BCz-TRZ-doped film emitted at 499 nm with a photoluminescence quantum yield (PLQY) of 50%. The optimized blue OLED exhibited a turn-on voltage (Von) of 3.2 V, a peak EQE of 11.9%, a maximum CE of 33.6 cd/A, and a maximum PE of 33.0 lm/W. In 2020, S. K. Jeon et al. investigated the luminescent properties of an exciplex system using DMAC-DPS (10,10′-(4,4′-sulfonyldi(4,1-phenylene))bis(9,9-dimethyl-9,10-dihydroacridine)), a material with TADF characteristics, as the donor, and PO-T2T as the acceptor. The authors proposed a novel approach to enhance the EQE_max_ of OLEDs by dispersing the exciplex within a TADF matrix. The DMAC-DPS:PO-T2T-doped film exhibited an emission wavelength of 480 nm. The emissive layer comprised a blend of the TADF material DMAC-DPS and the n-type material PO-T2T, forming an exciplex. By limiting the n-type material content to 1 wt%, the resulting device exhibited outstanding performance, achieving an EQE_max_ of 15.3%, a substantial improvement over conventional exciplex OLEDs with 50% n-type material content (EQE of 10.8%) [56]. Due to the limited energy gap between the HOMO and the LUMO, exciplexes in the deep-blue region exhibit lower EQE and poorer color purity, resulting in limited success for blue OLEDs based on exciplex emission [22]. Particularly in deep-blue emission, the demand for higher energy transitions poses greater challenges to the design of exciplex systems, often requiring further optimization of molecular structures to enhance the blueshift of the spectrum while maintaining an efficient RISC process. Additionally, the hot exciton mechanism serves as an effective strategy for achieving high-performance blue fluorescence. This process involves a transition between high-lying triplet states and singlet states, enabling efficient utilization of triplet excitons through RISC from T2 to S1. Table 1 presents a selection of hot exciton-based blue-emitting materials, compared alongside blue exciplex systems.

### 3.2. Exciplex as an Emitter in WOLEDs

**Figure 11:**
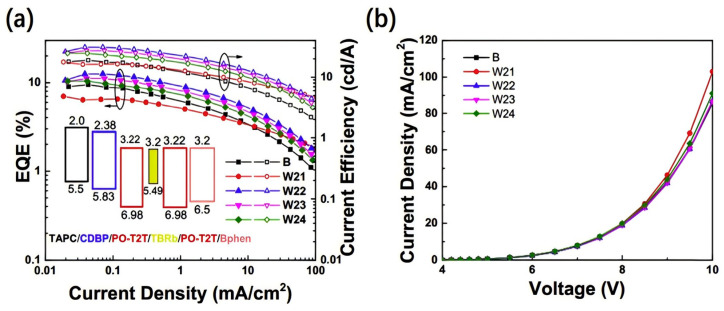
(**a**) EQE and CE−current density characteristics of device B, W21, W22, W23, and W24. Inset: the schematic energy level diagram of the devices. (**b**) Current density−voltage characteristics of the devices [63].

White organic light-emitting diodes (WOLEDs), which have shown immense potential for applications in solid-state lighting and flat-panel displays, have attracted significant attention in recent years, becoming a major focus of research. With advantages such as high luminous efficiency, wide color gamut, and low energy consumption, WOLEDs are demonstrating remarkable development potential in both commercial applications and academic research. In 2015, Duan et al. designed a highly efficient blue exciplex, TCTA: Bphen (4,7-Diphenyl-1,10-phenanthroline), which was combined with the yellow phosphorescent material of PO-01 to fabricate an F/P hybrid WOLED. By introducing a 3 nm-thick Bphen blocking layer, the excited states in the TCTA: Bphen/Bphen system were effectively confined at the interface, enabling efficient utilization of triplet excitons by the yellow emitting material through energy transfer, thereby enhancing exciton utilization efficiency. The resulting WOLED achieved a PE_max_ of 9.03 lm/W, an EQE_max_ of 4.3%, and a low turn-on voltage of approximately 3.0 eV, along with white light emission coordinates of (0.30, 0.35) [62]. In hybrid white OLEDs, single-emission-layer (S-EML) mixed WOLEDs have not yet been thoroughly explored. Additionally, finding suitable high T1 blue fluorescent materials for energy transfer remains a significant challenge. To address this, Lee et al. designed a blue exciplex based on (4,4′-Bis(carbazol-9-yl)-2,2′-dimethylbiphenyl) CDBP: PO-T2T as the blue fluorescent material, successfully fabricating an S-EML hybrid WOLED with low turn-on voltage and high efficiency. Without using light out-coupling techniques, the fabricated device achieved a remarkably low V_on_ of 2.5 V. Its CE_max_ and PE_max_ reached 67.0 cd/A and 84.1 lm/W, respectively, while the EQE_max_ achieved an impressive 25.5%. The device exhibited chromaticity coordinates of (0.40, 0.43). Lee et al. concluded that S-EML hybrid WOLEDs provide favorable conditions for fabricating large-area and low-cost devices through solution-processing methods [34]. The device based on the exciplex formed by CDBP: PO-T2T has demonstrated remarkable efficiency, attracting widespread attention. In 2018, He et al. successfully achieved a high-efficiency, non-doped WOLED by inserting an ultrathin yellow, fluorescent layer, 2,8-Di-tert-butyl-5,11-bis(4-tert-butylphenyl)-6,12-diphenyltetracene (TBRb), into the bilayer blue exciplex system formed by CDBP and PO-T2T (Figure 11). The final device achieved a CE_max_ of 27.2 cd/A, a PE_max_ of 21.4 lm/W, and an EQE_max_ of 11.2%, with CIE coordinates of (0.35, 0.39). By inserting the ultrathin layer, they found that the trapping of charges by the yellow dye could be suppressed, thereby enhancing the device’s efficiency [63]. In 2019, Ma et al. fabricated a high-efficiency, low-efficiency roll-off WOLED by inserting an ultrathin layer of yellow phosphorescent Bis(4-phenylthieno[3,2-c]pyridinato-N,C2′) (acetylacetonate) iridium(III) (PO-01) into the mixed EML of CDBP:PO-T2T. By inserting the yellow dye into the exciplex-based mixed layer, the phosphorescent layer was uniformly distributed in the exciton distribution zone, allowing excitons to transfer directly to the phosphorescent emitters, thereby reducing exciton quenching and improving exciton utilization. The high-efficiency WOLED proposed by Ma et al. achieved an EQE_max_ of 20.4%, with CE_max_ and PE_max_ values of 62.8 cd/A and 75.9 lm/W, respectively, and CIE coordinates of (0.42, 0.47). By broadening the exciton distribution region and actively improving exciton utilization, the strategy successfully enhanced WOLED efficiency and reduced efficiency roll-off [64].

**Figure 12:**
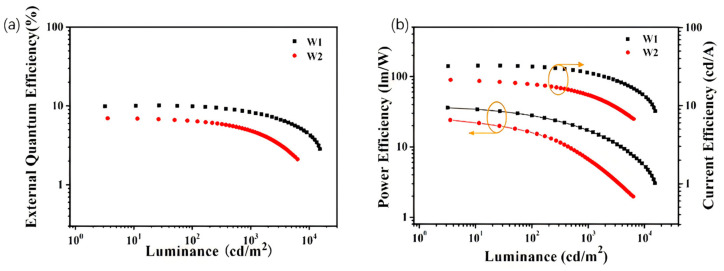
(**a**) External quantum efficiency versus luminance characteristics. (**b**) Current efficiency and power efficiency versus luminance characteristics [65].

Although two-color OLEDs exhibit high efficiency and stable spectra, their poor color purity and consistently low color rendering index (CRI), which is always below 80, limit their commercial applications. Therefore, further development of two-color WOLEDs with high CRI and high efficiency has become a key research focus. In 2017, Li et al. fabricated a complementary-color WOLED with high CRI by introducing Bis[2-(diphenylphosphino)phenyl]ether oxide (DPEPO) as a spacer, using mCP: PO-T2T as the blue exciplex and TAPC: PO-T2T as the orange exciplex. By increasing the number of spacers and optimizing their positions, the red and blue emissions were effectively balanced. The final WOLED achieved a CE_max_ of 16.2 cd/A, a PE_max_ of 11.3 lm/W, and an EQE_max_ of 7.92%, with CIE coordinates of (0.31 ± 0.00, 0.37 ± 0.02). Notably, its CRI reached 83, meeting commercial standards [5]. In 2019, Ma et al. developed a low-efficiency roll-off and high-efficiency fluorescent WOLED by inserting a hole-transporting layer (TCTA) into the blue exciplex 26DCzPPy: PO-T2T. The study revealed that introducing a TCTA interlayer into the EML formed a transitional interfacial exciplex with PO-T2T. The resulting green exciplex was capable of absorbing part of the energy from the blue exciplex for light emission and efficiently transferring the remaining energy to the yellow, fluorescent dye TBRb. This mechanism effectively reduced exciton quenching in 26DCzPPy: PO-T2T, improved exciton utilization, significantly lowered efficiency roll-off, and enhanced device efficiency. As shown in Figure 12, the resulting WOLED achieved CE_max_, PE_max_, and EQE_max_ of 32.6 cd/A, 35.9 lm/W, and 10.1%, respectively. Even at a brightness of 1000 cd/m², the device still maintained the efficiencies of 25.6 cd/A, 16.1 lm/W, and 8.1%. Furthermore, it exhibited a low V_on_ of only 2.8 V and a CIE coordinate of (0.36, 0.53). By introducing a specialized interlayer into the EML to effectively reduce efficiency roll-off, this approach provides a feasible solution for improving WOLED efficiency while offering valuable insights for future device design [65].

Electroplex emission, often considered to reduce device efficiency, is also associated with lowered color purity due to its red-shifted nature compared to monomer emission in OLED devices. In 2015, Chen and co-workers fabricated the TAPC:26DCzPPy exciplex emitter for organic light-emitting diodes (OLEDs) by blending the electron-rich hole-transporting material TAPC (1,1-bis[4-[N,N-di(p-tolyl)amino]phenyl]cyclohexane) with the bipolar acceptor material 26DCzPPy in a mixed ratio. Their investigation revealed that TAPC:26DCzPPy-based OLEDs exhibited mixed exciplex and electroplex emissions, resulting in white light emission with an exciplex peak at 450 nm and an electroplex peak at 585 nm. Devices utilizing this system as the emitting layer achieved a maximum EQE_max_ of 1.13% and a CE_max_ of 2.19 cd/A. This study elucidated the role of intermolecular CT interactions between electron-rich donors and bipolar acceptors in exciplex formation, offering valuable insights for developing efficient white or multicolor OLEDs, although the overall efficiency remains low and requires further enhancement [66]. In 2023, Zhang et al. successfully fabricated a highly efficient WOLED by combining exciplex emission with electromer emission to construct a bimolecular excited system. They first prepared the blue exciplex TAPC: TPBi, which exhibited a high-energy peak at 462 nm. Due to the recombination of neighboring electron-hole pairs, this material also displayed a low-energy peak at 586 nm under electrical excitation. To further improve device performance, they developed a green exciplex (4,4′,4″-Tris(N-3-methylphenyl-N-phenyl-amino)-triphenylamine) m-MTDATA: TPBi. Owing to the high T1 value of TAPC: TPBi, part of the energy could be transferred to m-MTDATA: TPBi for green light emission, while the remaining energy contributed to the blue light emission from TAPC: TPBi itself. The structure of the fluorescence-based WOLED, constructed on the bimolecular excited system, which did not contain fluorescent dyes, achieved a CE_max_ of 11.3 cd/A and a PE_max_ of 11.8 lm/W, with a low V_on_ of only 2.76 V. Remarkably, the device achieved CIE coordinates of (0.33, 0.38), which are very close to the ideal white light coordinates of (0.33, 0.33), and a CRI of 81, meeting commercial application standards. Zhang et al. noted that the TAPC: TPBi mixed layer played a critical role in the device, not only generating red and blue dual-color emissions simultaneously but also regulating exciton recombination in the EML, balancing carrier transport, improving device efficiency, and reducing V_on_ [67]. Table 2 summarizes the performance of exciplexes as emitters applied to WOLEDs as mentioned in Section 3.2.

## 4. Blue Exciplexes as Hosts and Cohosts in OLEDs

Frontier Molecular Orbitals (FMOs), including HOMO and LUMO, are key to understanding electron distribution and charge transfer in donor-acceptor (D/A) systems in OLEDs [19]. Their alignment boosts exciton generation and minimizes energy loss, enhancing device efficiency and stability [68]. Unlike TADF materials, in exciplex-based OLEDs, electrons and holes inject into acceptors’ LUMOs and donors’ HOMOs, respectively, followed by electron exchange between D/A FMOs. Exciplex hosts show strong bipolarity and low injection barriers, improving performance while retaining TADF effects.

The RISC in exciplex systems enables upconverted singlet excitons to transfer to dopants via Förster resonance energy transfer (rather than Dexter transfer), a more efficient pathway that reduces efficiency roll-off. Exciplex emission typically involves spectral broadening and red-shift, making high color purity challenging, thus rendering exciplexes more suitable as hosts than emitters [37,38,69,70,71].

For an exciplex to function as an effective host, it must meet specific conditions: (i) the triplet state (T1) of the exciplex should be lower than that of the individual D/A counterparts to confine the excitation energy within the exciplex; (ii) the exciplex T1 must be higher than that of the dopant to ensure emission originates from the dopant; and (iii) the emission of the exciplex should have adequate overlap with the dopant’s absorption spectrum to enable efficient energy transfer. This section gathers recent findings on the application of exciplex hosts or co-hosts in high-efficiency OLEDs [70,72,73].

### 4.1. Blue Exciplexes as Host in OLEDs

In OLEDs, the combination of a blue exciplex host with a blue phosphorescent dopant offers significant advantages for both efficiency and device stability. This host-dopant system leverages the high IQE of phosphorescent materials, enabling near-complete utilization of triplet excitons. The exciplex host, with its favorable triplet energy transfer properties, supports efficient triplet harvesting and minimizes non-radiative decay pathways, making it highly compatible with blue phosphorescent dopants.[74,75] Moreover, the exciplex-hosted phosphorescent structure not only enhances device lifetime but also provides improved color stability, addressing the challenges typically associated with the longevity and color purity of blue OLEDs [76].

**Figure 13:**
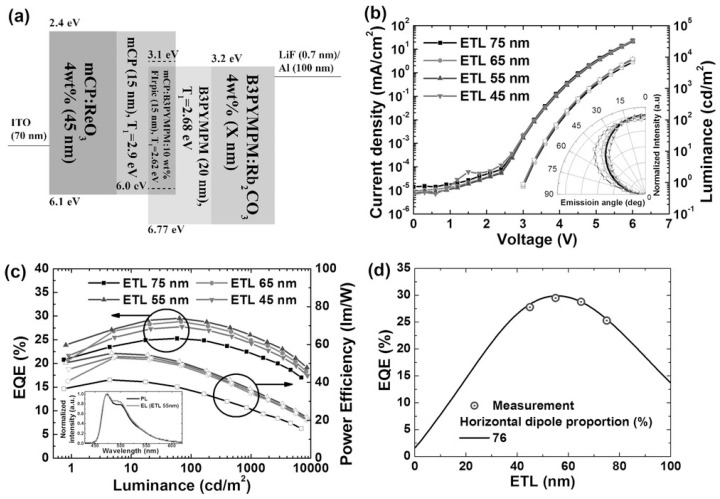
(**a**) Schematic diagram of the device structure and energy levels of the consisting layers by Kim et al. (**b**) J-V-L characteristics of the OLEDs according to the thickness of ETL. Inset: scatters = emission patterns of the EL intensity, solid line = Lambertian emission pattern. (**c**) EQE and power efficiency of the OLEDs. Inset is the normalized PL and EL intensity of ETL 55 nm device at 1000 nit. (**d**) Calculated EQEs as a function of the thickness of an ETL with experimentally measured maximum EQEs (circles) [77].

In this context, Kim et al. reported a breakthrough device design, utilizing mCP as a donor material and B3PYMPM as an acceptor material to form a blue exciplex host. By doping the blue phosphorescent dopant Bis[2-(4,6-difluorophenyl)pyridinato-C2,N](picolinato)iridium(III) (FIrpic) into the exciplex host, the device achieved a maximum EQE of 29.5% with a CE of 62.2 cd/A (Figure 13). The exciplex structure’s ability to confine triplet excitons effectively and support RISC allows both singlet and triplet excitons to contribute to emission. Additionally, the exciplex host showed a low driving voltage of 3 V and minimal efficiency roll-off, making it suitable for high-brightness applications [77].

**Figure 14:**
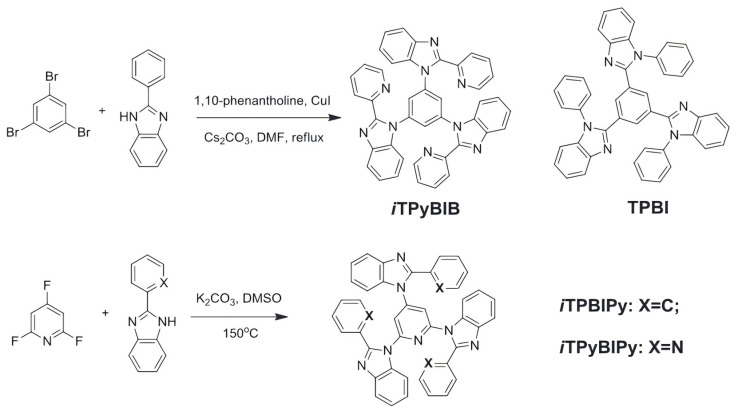
Chemical structure of TPBI and synthetic routes for the N-linked iTPyBIB, iTPBIPy, and iTPyBIPy [78].

Traditional electron donor materials such as mCP, TAPC, and TCTA have been widely used, but efficient electron acceptor materials in exciplex systems remain relatively scarce. To address this issue, Hu et al. designed and synthesized a novel benzoimidazole-containing electron acceptor material, 1,1′,1″-(pyridine-2,4,6-triyl)tris(2-(pyridin-2-yl)-1H-benzo[d]imidazole) iTPyBIPy, through a simple one-step C–N coupling reaction (Figure 14). They introduced an isomeric N-linkage into iTPyBIPy, which significantly simplified the synthesis process. Using mCP as the donor material and FIrpic as the dopant, the fabricated blue phosphorescent OLED exhibited excellent performance with a maximum efficiency of 38.5 cd/A, 36.9 lm/W, and an EQE_max_ of 19.3%, along with a satisfactory CIE color coordinate of (0.15, 0.29). This work fills the gap in the application of benzoimidazole-containing electron acceptor materials in exciplex-based devices [78]. Lee et al. first applied the high-triplet-energy (N,N-bis(4-(tert-butyl)phenyl)-3,5-bis(3,6-di-tert-butyl-9Hcarbazol-9-yl)aniline) t-DCDPA: DBFTrz (2,8-bis(4,6-diphenyl1,3,5-triazin-2-yl)dibenzo[b,d]furan) exciplex host in deep-blue PhOLEDs, achieving an EQE of 16.4% and color coordinates of (0.14, 0.19) with tris[2,6difluoro-3-(pyridin-2-yl)benzonitrile]iridium(III) (FCNIr) as the emitter, pioneering the use of exciplex hosts in deep-blue phosphorescent devices and providing a key foundation for further studies [79]. In the same year, Kim et al. employed a high-triplet-energy mCP:BM-A10 exciplex host (T1 > 2.8 eV) with FCNIr as the emitter, achieving a deep-blue PhOLED with an EQE of 24%, CIE coordinates of (0.15, 0.21), and a turn-on voltage of 2.9 V, with a lifetime markedly surpassing single-host systems, highlighting the advantages of exciplex hosts in enhancing deep-blue device performance [13]. In 2018, Lee et al. generated an exciplex by mixing 2,2′-Di(9H-carbazol-9-yl)biphenyl (oCBP) and 9-(3′-(9Hcarbazol-9-yl)-5-cyano-[1,1′-biphenyl]-3-yl)-9H-carbazole-3-carbonitrile (CNmCBPCN) host materials, with a triplet energy of 2.94 eV and a singlet energy of 3.30 eV. This exciplex host achieved an external quantum efficiency of 18.8% in blue phosphorescent OLEDs and extended the lifetime by more than 1.8 times [80].

Although the exciplex co-host system stands out as a highly promising approach for achieving high-performance phosphorescent OLEDs, further improvements are still needed to achieve blue OLEDs with excellent color purity. In 2020, Chen et al. successfully constructed an exciplex co-host system formed by the donor of mCP and the acceptor of 4,6-Bis(3,5-di(pyridin-4-yl)phenyl)-2-phenylpyrimidine (B4PyPPM). Compared to traditional host-based devices using mCP, the blue phosphorescent OLEDs based on the exciplex co-host exhibited stable color purity and a higher EQE of 20.1%. The result demonstrated the potential of the co-host system as an effective strategy for developing simplified OLEDs with outstanding performance, suitable for future solid-state lighting and display applications [81]. Although several exciplex hosts for blue phosphors have been reported, only a limited number can effectively harvest triplet excitons from pure-blue phosphors, and none have demonstrated the ability to guarantee lifetime. Therefore, in the same year, Kim et al. utilized an mCBP: SiCzTrz (9-(4-Phenyl-6-(3-(triphenylsilyl)phenyl)-1,3,5-triazin-2-yl)-9H-carbazole) electroplex host with Ir(cb)₃ as the dopant to develop pure-blue PhOLEDs, achieving an EQE of 27.6%, an LT50 exceeding 10,000 h at 100 cd/m², and CIE coordinates of (0.12, 0.13), underscoring the superiority of electroplex hosts for high-efficiency, long-lifetime pure-blue devices [74].

Extensive research has enabled high-efficiency PhOLEDs with single exciplex hosts. However, enhancing efficiency at high luminance remains a key challenge, limiting applications in solid-state lighting and bright displays [31]. In 2021, Ma et al. utilized dual exciplex hosts mCBP: PO-T2T and mCBP: B4PyPPM for the FIrpic emitter, markedly enhancing blue PhOLED performance with an EQE of 25.61% and a CE of 45.77 cd/A, while effectively reducing roll-off at high luminance, demonstrating the potential of dual-host strategies in optimizing exciplex-based OLEDs [82]. In the same year, Vasilopoulou et al. combined a low triplet energy HTM (T2FQ/TQ2F) with a (bis(diphenylphosphine oxide)dibenzofuran) DBFPO/TSPO1 (diphenyl[4-(triphenylsilyl)phenyl]phosphine oxide) interface exciplex to confine excitons, achieving efficient blue TADF OLEDs with a 2.5 V turn-on voltage, an EQE of 41.2%, and 34.8% at 1000 cd/m² with low roll-off, underscoring the potential of device engineering in enhancing blue OLED performance [83].

Blue PhOLEDs have been regarded as a highly promising alternative to blue FOLEDs. However, previously reported blue PhOLEDs with CIE y coordinates below 0.2 have exhibited LT_70_ values (the time to reach 70% of the initial brightness) of less than 100 h at an initial brightness (L_0_) of 1000 cd/m², a performance insufficient for practical applications [84]. Kim et al. (2022) utilized a (9-(3-(triphenylsilyl)phenyl)-9H-3,9′-bicarbazole) SiCzCz: SiTrzCz2 (9,9′-(6-(3-(Triphenylsilyl)phenyl)-1,3,5-triazine-2,4-diyl)bis(9H-carbazole)) exciplex host and optimized PtON-TBBI (BD-02) dopant to develop deep-blue PhOLEDs, achieving an EQE of 25.4%, a CIE y = 0.197, and an LT70 of 1113 h at 1000 cd/m², significantly improving color purity and lifetime, and demonstrating a breakthrough in exciplex-hosted deep-blue phosphorescent devices [85]. In 2024, Li et al. developed a deep-blue PhOLED using a (9-(3-(triphenylsilyl)phenyl)−9H-3,9-bicarbazole) SiBCz:SiTrzCz2 exciplex host and PtON5N-dtb dopant, achieving a peak EQE of 20.4%, retaining 18.5% at 1000 cd/m², with an LT90 of 85 h and CIE coordinates of (0.13, 0.17), highlighting the exceptional potential of exciplex hosts in low-roll-off, long-lifetime deep-blue devices [86]. Recently, Jang Hyuk Kwon reported n-type hosts CNCzPyDFCN and CNDFPyCzCN (T1 > 2.96 eV), designed via simulation and paired with oCBP as exciplex hosts for blue PhOLEDs. The oCBP:CNCzPyDFCN host achieved an EQE of 29% and LT50 of 33.9 h at 1000 cd/m², demonstrating excellent stability, whereas oCBP:CNDFPyCzCN reached 26.6% EQE but only 8.2 h lifetime, underscoring the critical role of exciplex design in blue device performance [87].

### 4.2. WOLEDs Based on Blue Exciplexes as Hosts

**Figure 15:**
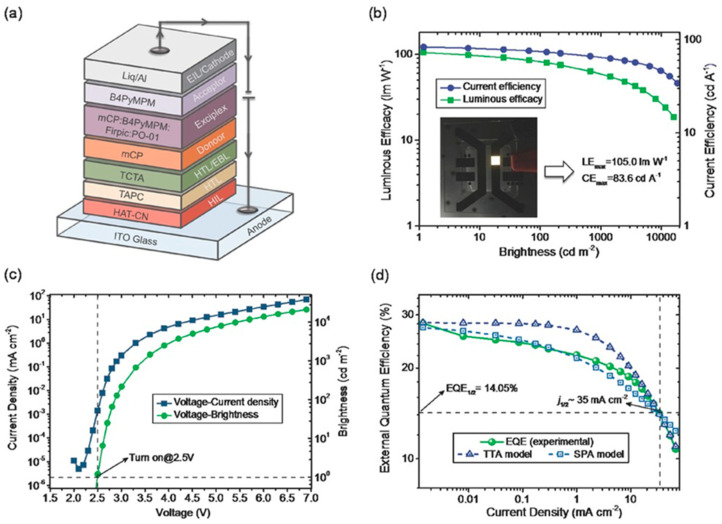
(**a**) Device structure of the white OLED. (**b**) Luminous efficacy–current efficiency–brightness characteristics. The inset is the image of the corresponding white OLED. (**c**) Current density–voltage−brightness characteristics. (**d**) External quantum efficiency−current density curve. The green solid line is the measured data, while the two blue dashed lines are the EQE fitted upon considering TTA and SPA models [90].

Although several high-performance blue OLEDs based on exciplex host systems have been reported, research on the development of WOLEDs using exciplex hosts remains relatively limited. In 2016, Ma et al. utilized the mCP:B3PYMPM exciplex host with FIrpic as the blue phosphor and ultrathin PO-01 as the yellow layer to develop a structurally simple WOLED, achieving an EQE of 20.0%, a CE of 64.5 cd/A, and a low turn-on voltage of 2.4 V, highlighting the potential of exciplex hosts in designing efficient white light devices [88]. To meet the requirements for lighting applications, the PE generally needs to exceed 100 lm/W, yet the lack of suitable out-coupling enhancement devices remains a challenge to overcome [89]. In 2017, Fung et al. utilized an mCP:B4PyMPM (4,6-Bis(3,5-di(pyridin-4-yl)phenyl)-2-methylpyrimidine) exciplex host (T1 = 2.75 eV) to design a single-EML hybrid WOLED with FIrpic and PO-01 as emitters. As shown in Figure 15, the device achieved a PE of 105.0 lm/W, EQE of 28.1%, and CIE (0.40, 0.48), with a predicted PE up to 210 lm/W, demonstrating the potential of exciplex hosts in efficient, energy-saving white light devices [90]. To bring WOLED devices into practical production, it is essential to ensure not only sufficient efficiency but also excellent color stability. Sheng et al. developed a WOLED using the mCP:B4PyPPM exciplex co-host with FIrpic and PO-01 dopants. The resulting WOLED achieved an EQE of 21.7%, a CE of 66.4 cd/A, and a Von of 2.5 V, with a CIE shift of only (0.008, 0.002) over 4-7 V, highlighting the potential of exciplex hosts in efficient and spectrally stable white light devices [81].

**Figure 16:**
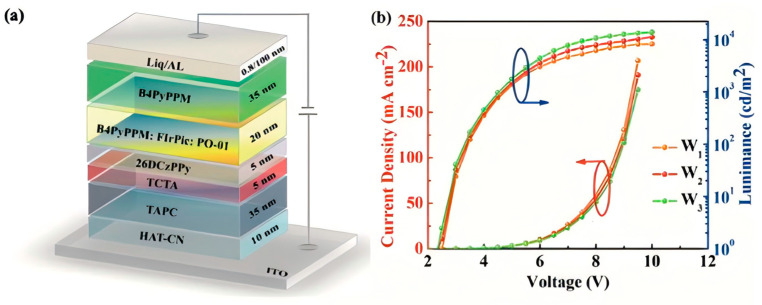
(**a**) The structure of the WOLEDs. (**b**) The current density−voltage−luminance characteristics of devices W_1_–W_3_ [92].

In the formation of co-host systems, it is commonly observed that high efficiency is often accompanied by a decline in color quality. Therefore, developing a device that achieves both high efficiency and a good color gamut is a challenge worth exploring. In 2019, Liao et al. harnessed the 26DCzPPy:B4PyMPM exciplex co-host, integrating FIrpic and PO-01 dopants, to craft a WOLED. At a 4.0% PO-01 doping level, the device delivered an EQE of 27.3%, a CE of 79.0 cd/A, and a PE of 89.0 lm/W, with a mere 2.7 V turn-on voltage. Exhibiting minimal green emission and an impressive color gamut, this approach underscores the distinctive potential of exciplex systems for high-performance white light applications [91]. Leveraging a 26DCzPPy/B4PyPPM interfacial exciplex, Sheng et al. engineered a single-EML WOLED that, with excellent energy alignment and low PO-01 doping to curb Dexter transfer, attained an EQE of 23.1%, a CE of 81.1 cd/A, and a PE of 101.9 lm/W at an ultra-low 2.4 V turn-on voltage, alongside CIE coordinates of (0.45, 0.49), signaling a forward-looking trend in efficient, simplified white light device design (Figure 16) [92]. Recently, Xie et al. harnessed a Trz-PhCz:B4PyMPM exciplex co-host to integrate green, yellow, orange, and red phosphorescent emitters into OLEDs, yielding EQE values from 25.0% to 32.5%, while employing mCBP:B4PyMPM raised the blue OLED EQE to 26.3%; by skillfully blending blue and yellow EMLs, a two-color WOLED driven at 2.2 V delivered a PE of 137.4 lm/W and EQE of 36.9%, sustaining high efficiency at 1000 cd/m², underscoring the utility of exciplex systems in multicolored, high-performance white light devices [93].

**Figure 17:**
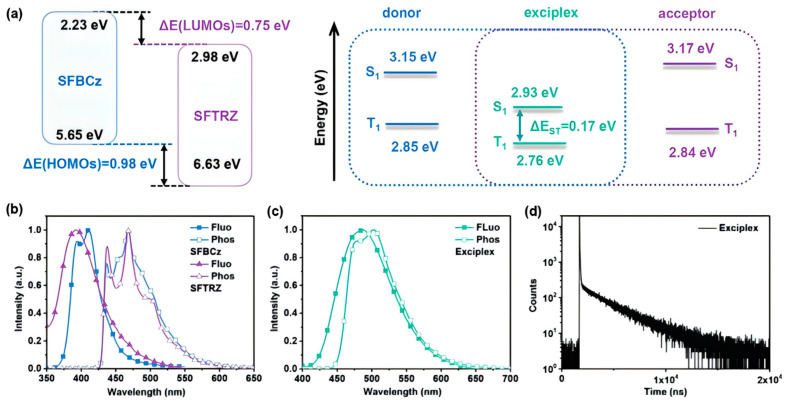
(**a**) The energy levels of HOMO, LUMO, S1, and T1 of SFBCz, SFTRZ, and exciplex. (**b**) The fluorescence and phosphorescence spectra of SFBCz and SFTRZ. (**c**) The fluorescence and phosphorescence spectra of the exciplex-forming host. (**d**) The PL decay curve of the exciplex-forming host recorded at the emission peak [69].

Recently, the strategy of using blue exciplexes as hosts combined with TADF materials to improve device efficiency and stability has attracted significant attention. Researchers have explored applying TADF dopants within blue exciplex-based host systems, leveraging the advantages of both: exciplexes offer a broad emission spectrum and charge balance capability, while TADF emitters provide efficient exciton harvesting. Incorporating TADF materials into exciplex hosts enables the effective utilization of singlet and triplet excitons. Currently, TADF OLED devices using exciplexes as the primary emitters, especially S-EML WOLEDs employing TADF-sensitized fluorescence (TSF), remain relatively scarce. Due to the lack of stable blue exciplex compounds, such devices often face issues such as low blue light efficiency and significant efficiency roll-off [15]. In 2020, Duan et al. introduced an innovative cyan exciplex, 9-(9,9′-spirobi[fluoren]-3-yl)-9′-phenyl-9H,9′H3,3′-bicarbazole (SFBCZ): SFTRZ (2-(3′-(9,9′-spirobi[fluoren]-3-yl)-[1,1′biphenyl]-3-yl)-4,6-diphenyl-1,3,5-triazine), utilizing bipolar large-volume π-spaced groups to widen the D-A subunit distance, markedly extending blue-shift emission while refining charge transport properties (Figure 17). By channeling excitons through multiple energy funnel processes within a broad charge-complex region, this approach mitigated exciton annihilation at high current densities, substantially enhancing the lifespan and stability of WOLEDs [69]. The authors employed the SFBCZ:SFTRZ exciplex as the central emitter, integrating it with the blue TADF sensitizer 2,3,4,5,6-pentakis-(3,6-di-tert-butyl-9H-carbazol-9-yl)benzonitrile (5TCzBN) and yellow fluorescent dopant TBRb to construct a single-layer warm white OLED, which delivered an EQE of 21.4% and PE of 69.6 lm/W at a low driving voltage of 3.08 V, with a T80 surpassing 8200 h, highlighting the potential of combining exciplexes and TADF for efficient, durable white light devices. Chen et al. (2022) engineered a single-layer WOLED with a 26DCzPPy:PO-T2T interfacial exciplex and 10-(4-(4,6-diphenyl-1,3,5-triazin-2-yl)phenyl)-10H-spiro[acridine-9,9′-fluorene] (SpiroAC-TRZ) sensitizer, operating at a low turn-on voltage of 2.7 V, yielding a CE of 52.4 cd/A and PE of 60.9 lm/W, with efficiency roll-off limited to 14.5% at 500 cd/m², marking a breakthrough for ultrathin-layer structures in low-roll-off white light devices [94]. In the same year, Wang et al. capitalized on a high-T1 (3.15 eV) mCP/mSiTRZ (2-Phenyl-4,6-bis(3-(triphenylsilyl)phenyl)-1,3,5-triazine) interface exciplex combined with rapid-RISC (1.15 × 10^7^ s^−1^) 5CzTRZ to produce a blue OLED, securing a peak EQE of 28.1%, sustained at 27.32% under 1000 cd/m² luminance; they further crafted an all-TADF WOLED by sensitizing 5, 10-bis(4-(9,9- dimethylacridin-10(9H)-yl)-2,6-dimethylphenyl)5,10-dihydroboran-threne (dmACDBA) with 5CzTRZ, yielding a driving voltage of just 3.1 V, an EQE of 27.3%, and a PE of 70.4 lm/W, exemplifying remarkable advances in efficiency and stability for solution-processed white light devices [49].

**Figure 18:**
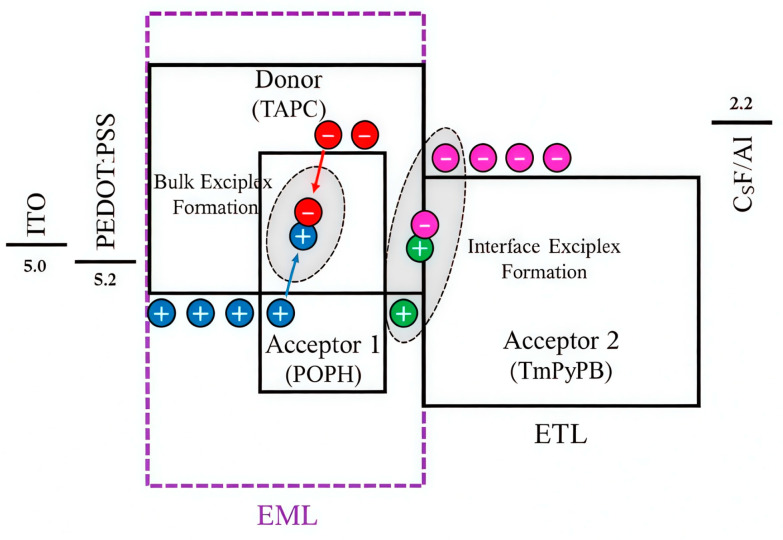
Proposed formation of the dual exciplexes (The blue and red circles represent the holes and electrons forming bulk exciplexes, respectively, while the green and pink circles represent the holes and electrons forming interface exciplexes, respectively.) [33].

Although interface exciplexes are considered suitable for use in solution-processed OLEDs, their exciton recombination region is confined to the interface between the EML and ETL. Without precise control of the EML thickness to facilitate effective energy transfer to the dopants, this limitation can result in the wastage of holes and electrons, leading to low device efficiency and severe efficiency roll-off. In 2023, Chen et al. employed solution processing to construct a TAPC:POPH (T1=3.01 eV) bulk exciplex and a TAPC/TmPyPB (1,3,5-Tri[(3-pyridyl)-phen-3-yl]benzene) (T1=3.13 eV) interface exciplex (Figure 18), paired with the 10-(4-(4,6-Diphenyl-1,3,5-triazin-2-yl)phenyl)-9,9-dimethyl-9,10-dihydroacridine (DMAC-TRZ) emitter, setting a record EQE of 26.7% for solution-processed blue TADF OLEDs; subsequent doping with Bis(2-phenylquinoline)(2,2,6,6-tetramethylheptane-3,5-dionate)iridium(III) (Ir(DPM)PQ2) produced a hybrid white OLED with an EQE of 24.1% and nearly voltage-invariant spectra, revealing the transformative potential of dual-exciplex designs for efficient and stable white light devices [33].

**Figure 19:**
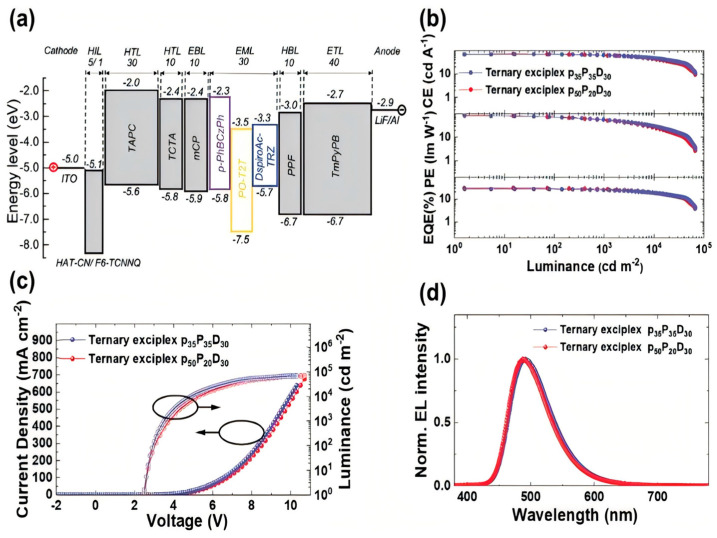
(**a**) Energy level diagram, (**b**) CE/ PE/ EQE−Luminance (CE/ PE/ EQE−L), (**c**) current density−voltage−luminance (J−V−L) characteristics, and (**d**) EL spectra of the devices based on ternary exciplexes p_35_P_35_D_30_ and p_50_P_20_D_30_ as EML [95].

In recent years, multi-resonance thermally activated delayed fluorescence (MR-TADF) materials have gradually become a research hotspot in OLED devices due to their high efficiency, pure color purity, and simple design. In particular, combining MR-TADF materials with exciplexes to fabricate OLED devices has emerged as a frontier in this field. In 2024, Su et al. successfully combined a (3,3′-(2,5-dimethyl-1,4-phenylene) bis(9-phenyl-9Hcarbazole) PhBCzPh: PO-T2T: DspiroAc-TRZ ternary exciplex (PLQY 72%, kRISC 2.0 × 10^7^ s^−1^) with the efficient MR-TADF material 2TCzBN to produce a sky-blue OLED, as shown in Figure 19. The device operated at a mere 2.5 V driving voltage, delivering a CE of 65.9 cd/A and an EQE of 36.2% with minimal roll-off; by blending blue and orange emission units, they achieved a warm white dual-EML OLED with an EQE exceeding 30%, underscoring the remarkable promise of ternary exciplex systems for narrow-band, high-efficiency white light devices [95]. Table 3 summarizes the performance of all exciplex-based host devices in Section 4.

## 5. Conclusions

This review systematically examined the dual roles of exciplexes as emitters and hosts: as emitters, blue exciplexes achieve high color purity and efficiency through optimized molecular design, outperforming traditional fluorescent and phosphorescent materials; as hosts, their wide energy bands and high triplet energy enable efficient exciton utilization and stable energy transfer, significantly advancing the performance of sky-blue, deep-blue, and white OLEDs. Consequently, OLEDs based on blue exciplexes exhibit exceptional potential in efficiency, turn-on voltage, and stability. However, challenges such as a limited deep-blue gamut, degradation induced by high-energy excitons, and spectral red-shift persist, impeding color purity and lifetime from meeting commercial standards.

Although blue exciplex designs have made strides in expanding exciton distribution and energy transfer, further improvements in emission efficiency and longevity are still required. Therefore, future research must delve deeper into the photophysical processes of blue exciplexes and develop new material systems with high PLQY and stability to address current limitations, thereby hastening their practical implementation in display and lighting applications.

## Figures and Tables

**Table 1:** Performance summary of blue exciplexes as emitters in blue OLEDs.

Exciplex System (EML)	EQE (%)	PE (lm/W)	CE (cd/A)	λ_EL_ (nm)	λ_PL_ (nm)	Von (V)	FWHM (nm)	CIE Coordinate	S1/T1 (eV)	Type	Ref
NPB:TPBi	3.7	2.6	2.8	450	-	2.5	-	(0.15, 0.13)	2.85/2.80	Exciplex	[35]
TCTA:B3PYMPM	10.0	-	-	495	484	2.8	-	-	-/-	Exciplex	[39]
mCP:PO-T2T	8.0	18.4	15.5	471	473	2.0	-	(0.17, 0.23)	-/-	Exciplex	[40]
NPB:DPTPCz	0.6	1.2	1.4	491	-	-	83	(0.25, 0.41)	-/-	Exciplex	[28]
CDBP:PO-T2T	13.0	27.8	26.6	-	476	2.5	77	(0.17, 0.29)	2.89/2.86	Exciplex	[34]
mCBP:PO-T2T	7.66	17.78	15.08	-	473	-	-	(0.17, 0.23)	-/-	Exciplex	[41]
CzSi:PO-T2T	6.1	7.0	8.9	465	-	3.0	-	(0.16, 0.21)	-/-	Exciplex	[42]
mCP:PO-T2T	16.0	26.4	27.0	480	-	3.0	-	(0.16, 0.28)	-/-	Exciplex	[42]
mCPPO1:PO-T2T	6.5	8.0	9.4	480	-	3.0	-	(0.18, 0.29)	-/-	Exciplex	[42]
TCTA:CzTrz	12.62	27.48	-	490	-	-	85	(0.27, 0.53)	2.85/2.85	Exciplex	[43]
CPF:PO-T2T	9.5	22.9	21.9	-		2.2	-	(0.17, 0.29)	-/-	Exciplex	[44]
CPTBF:PO-T2T	12.5	33.2	27.5	-		2.2	-	(0.18, 0.31)	-/-	Exciplex	[44]
TPAPB:TPBi	7.0	9.1	7.2	468	471	3.2	-	(0.14, 0.18)	2.66/2.61	Exciplex	[45]
TAPC:10% BFPD	7.8	-	21.7	508	488	4.6	-	-	2.88/2.84	Exciplex	[46]
CN-Cz2:PO-T2T	16.0	47.5	37.8	480	-	2.3	-	(0.20, 0.40)	-	Exciplex	[47]
mCP:24iPBIOXD	0.65	0.66	0.8	-	-	3.8	-	(0.19, 0.20)	-/-	Exciplex	[48]
mCP: iTPBIOXD	0.63	0.74	0.9	-	-	3.8	-	(0.20, 0.23)	-/-	Exciplex	[48]
mCP:8% HAP-3FDPA	10.2	-	-	437	433	4.0	87	(0.16, 0.12)	-/-	Exciplex	[24]
26DCzPPy:TB-3tBuCz	14.6	3.1	6.6	425	-	6.5	46	(0.158, 0.052)	3.07/3.07	Exciplex	[50]
mCBP:DNPhB	4.83	2.2	3.04	444	444	3.7	-	(0.152, 0.075)	3.27/3.25	Exciplex	[22]
NPB:TAZ	-	0.36	0.75	440		3.6	63	-	-/-	Exciplex	[51]
TCTA:BOBTFB	3.49	-	4.8	469	478	2.8	78	(0.15, 0.18)	2.92/2.78	Exciplex	[52]
P6:TCTA	9.1	-	-	433	-	6.0	101-122	-	2.93/2.92	Exciplex	[53]
DMAC-DPS:T2T	4.44	-	-	480	480	-	-	(0.20, 0.37)	-/-	Exciplex	[54]
DMAC-DPS: B4PyMPm	4.40	-	-	493	493	-	-	(0.24, 0.42)	-/-	Exciplex	[54]
TrisPCz:BCz-TRZ	11.9	33.0	33.6	-	499	3.2	-	(0.26, 0.50)	-/-	Exciplex	[55]
DMAC-DPS:1% PO-T2T	15.3	-	22.0	480	490	6.9	-	(0.20, 0.41)	-/-	Exciplex	[56]
TPA-PPI	5.02	6.13	5.66	434	440	-	-	(0.15, 0.11)	-/-	Hot Exciton Materials	[57]
TPMCN	2.18	1.96	2.69	463	445	3.5	-	(0.15, 0.18)	-/-	Hot Exciton Materials	[58]
Py-BPA-BPI	5.64	10.5	10.9	471	469	2.15	-	(0.17, 0.29)	-/-	Hot Exciton Materials	[59]
TPEPO	6.62	10.9	15.86	484	490	4.0	-	-	-/-	Hot Exciton Materials	[60]
TPP-TXO_2_	10.5	-	11.1	-	437	3.1	-	(0.152, 0.065)	-/-	Hot Exciton Materials	[61]

**Table 2:** Performance summary of blue exciplexes as emitters in WOLEDs.

Exciplex System (EML)	EQE (%)	PE (lm/W)	CE (cd/A)	V_on_ (V)	Ref
TCTA:Bphen+ PO-01	4.3	9.03	3.6	3.0	[62]
CDBP:PO-T2T (WOLED)	25.5	84.1	67.0	2.5	[34]
CDBP:PO-T2T + TBRb	11.2	21.4	27.2	-	[63]
CDBP:PO-T2T + PO-01	20.4	75.9	62.8	-	[64]
mCP:PO-T2T + TAPC:PO-T2T	7.92	11.3	16.2	-	[5]
26DCzPPy:PO-T2T + TBRb	10.1	35.9	32.6	2.8	[65]
TAPC:26DCzPPY	1.13	-	2.19	-	[66]
TAPC:TPBi + m-MTDATA:TPBi	-	11.8	11.3	2.76	[67]

**Table 3:** Performance summary of blue exciplexes as hosts in OLEDs.

Exciplex System (EML)	EQE (%)	PE (lm/W)	CE (cd/A)	λ_EL_ (nm)	V_on_ (V)	Type	Ref
mCP:B3PYMPM:FIrpic	29.5	55.4	62.2	-	3.0	Sky-Blue Host	[77]
mCP:iTPyBIPy:FIrpic	19.3	36.9	38.5	-	3.2	Sky-Blue Host	[78]
t-DCDPA:DBFTrz:FCNIr	16.4	-	25.2	-	-	Deep-Blue Host	[79]
mCP:BM-A10:FCNIr	24.0	26.0	-	455	2.9	Deep-Blue Host	[13]
oCBP:CNmCBPCN	18.8	30.3	32.8	420	-	Deep-Blue Host	[80]
mCP:B4PyPPM:FIrpic	20.1	47.4	40.7	470	2.6	Sky-Blue Host	[81]
mCBP:SiCzTrz:Ir(cb)3	27.6	-	-	471	-	Pure-Blue Host	[74]
mCBP:PO-T2T + mCBP:B4PyPPM:FIrpic	25.61	54.31	45.77	470	-	Sky-Blue Host	[82]
DBFPO:TSPO1+TDBA-DI	41.2	87.2	72.2	458	2.5	Deep-Blue Host	[83]
SiCzCz:SiTrzCz2:BD-02	25.4	25.0	30.4	-	2.8	Deep-Blue Host	[85]
SiBCz:SiTrzCz2:PtON5N-dtb	20.4	-	-	470	2.7	Deep-Blue Host	[86]
oCBP:CNCzPyDFCN+CN–Ir	29.2	-	46.8	463	2.7	Blue Host	[87]
oCBP:CNDFPyCzCN+ CN–Ir	26.6	-	44.0	463	2.7	Blue Host	[87]
mCP:B3PYMPM:FIrpic + PO-01	20.0	75.3	64.5	-	2.4	WOLED Host	[88]
mCP:B4PyMPM:FIrpic + PO-01	28.1	105.0	83.6	-	-	WOLED Host	[90]
mCP:B4PyPPM:FIrpic + PO-01	21.7	73.6	66.4	-	2.5	WOLED Host	[81]
26DCzPPy:B4PyMPM:FIrpic + PO-01	27.3	89.0	79.0	-	2.7	WOLED Host	[91]
26DCzPPy/B4PyPPM:FIrpic + PO-01	23.1	101.9	81.1	-	2.4	WOLED Host	[92]
Trz-PhCz:B4PyMPM + mCBP:B4PyMPM	36.9	137.4	-	-	2.2	WOLED Host	[93]

## Data Availability

No new data were created or analyzed in this study. Data sharing is not applicable to this article.

## Abbreviations

The following abbreviations are used in this manuscript:

CE	Current efficiency
CIE	Commission Internationale de l’Éclairage
CT	Charge transfer
ΔE_ST_	Singlet-triplet energy gap
EQE	External quantum efficiency
FMO	Frontier Molecular Orbital
HOMO	Highest Occupied Molecular Orbital
IQE	Internal quantum efficiency
LUMO	Lowest Unoccupied Molecular Orbital
MDPI	Multidisciplinary Digital Publishing Institute
MR-TADF	Multi-resonance thermally activated delayed fluorescence
NTSC	National Television System Committee
OLED	Organic light-emitting diode
PE	Power efficiency
PhOLED	Phosphorescent organic light-emitting diode
PLQY	Photoluminescence quantum yield
RISC	Reverse intersystem crossing
TADF	Thermally activated delayed fluorescence
TRPL	Time-Resolved Photoluminescence
TTET	Triplet-triplet energy transfer
TTF	Triplet-triplet fusion
WOLED	White organic light-emitting diode
−ΔGcs	Driving force
TSF	TADF-sensitized fluorescence
FWHM	Full width at half maximum
